# Supplementary figures and images for: An isoform-specific function of Cdc42 in regulating mammalian Exo70 during axon formation
Source: Life Sci Alliance. 2022 Dec 21;6(3):e202201722. doi: 10.26508/lsa.202201722 (PMC9772827; doi:10.26508/lsa.202201722)

Figure 2B

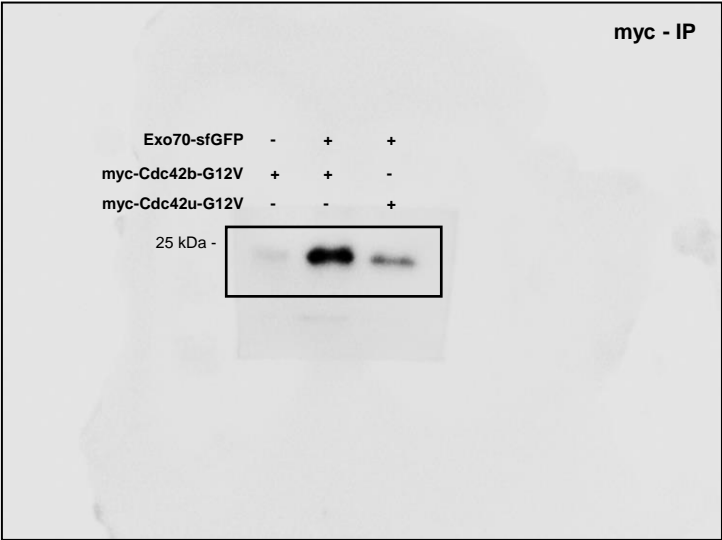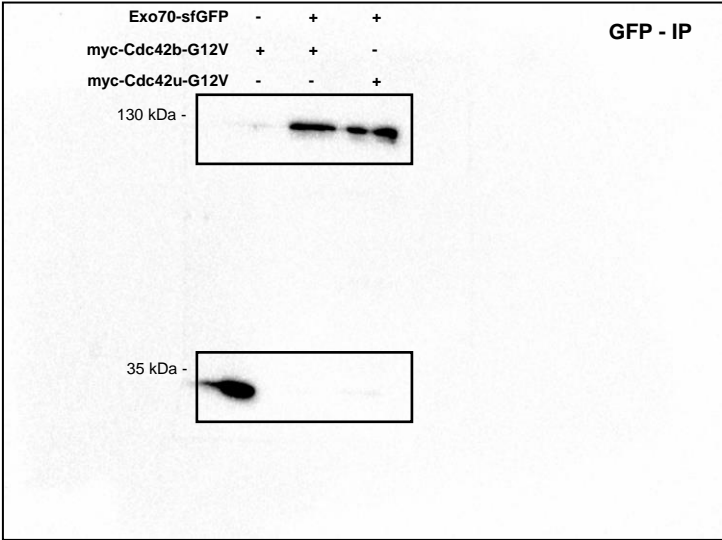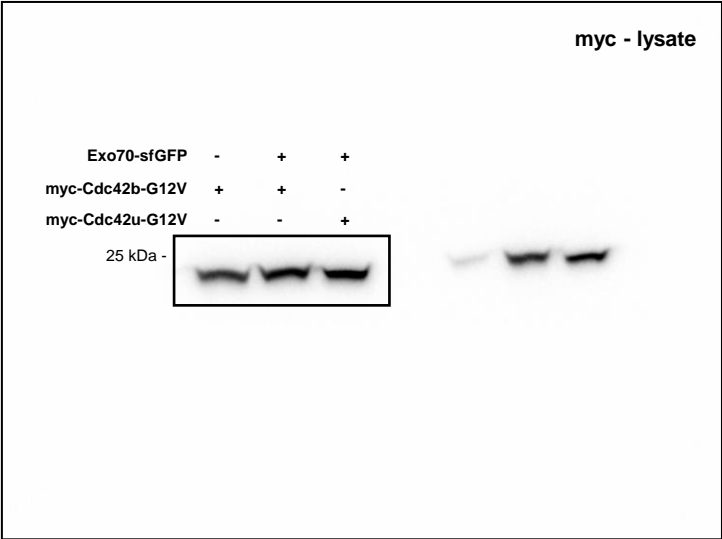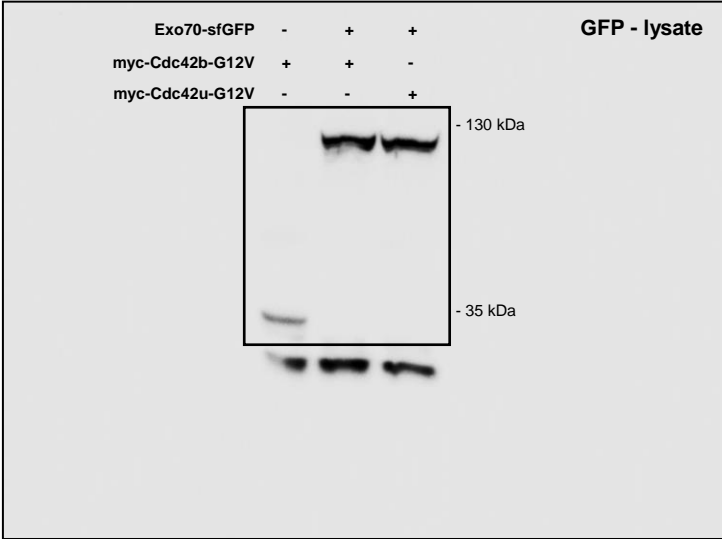

Figure 2C

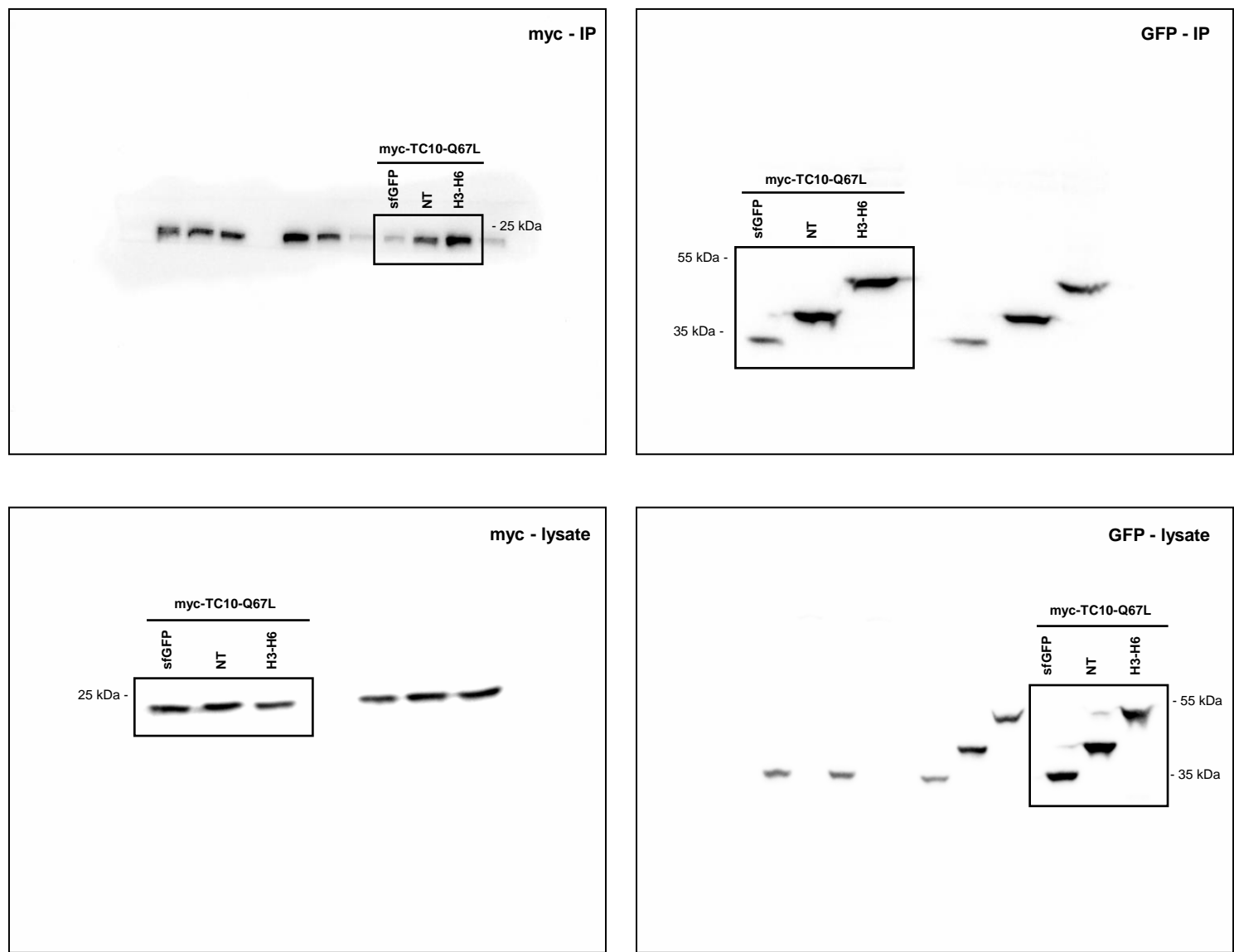

Figure 2D

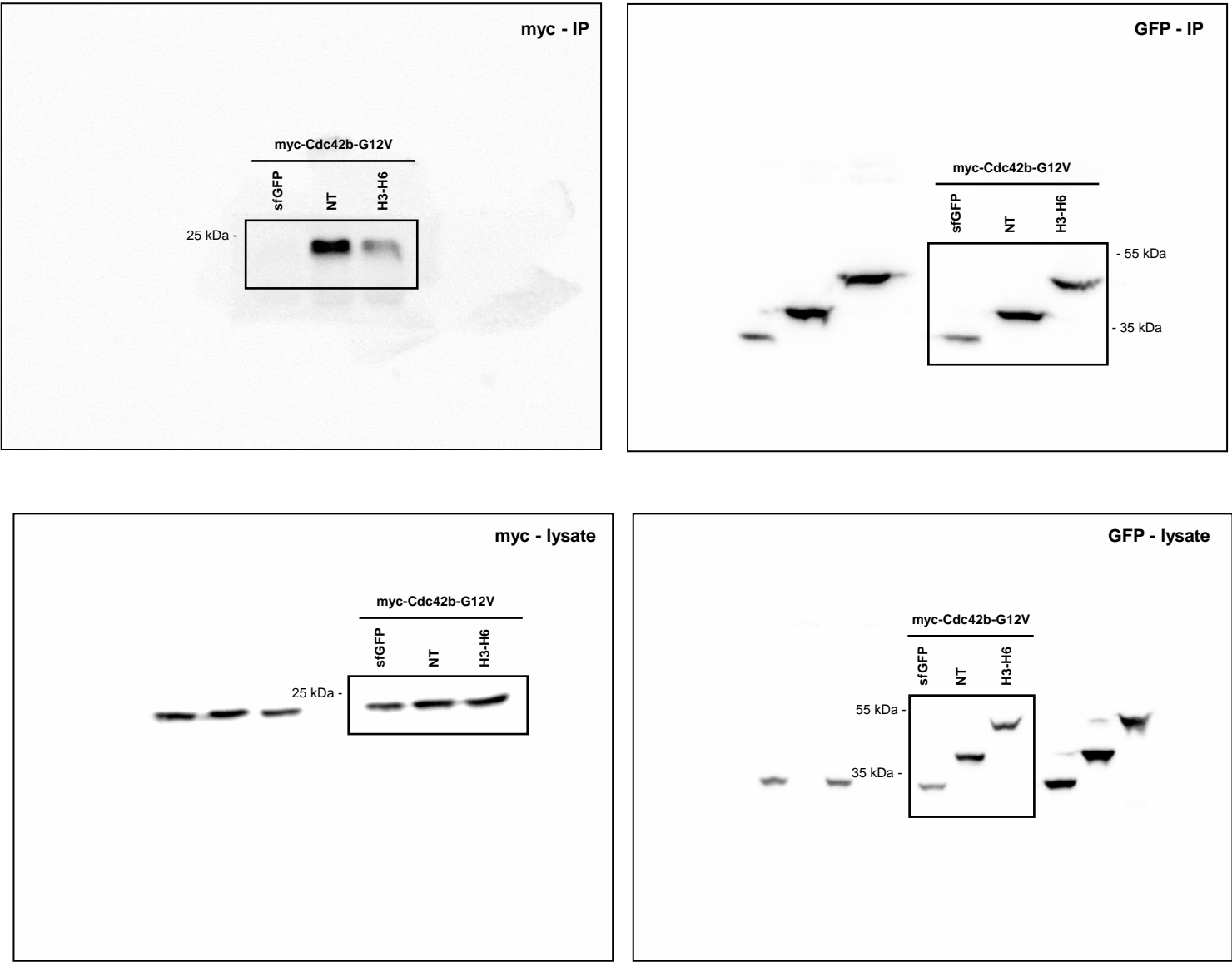

Figure 2E

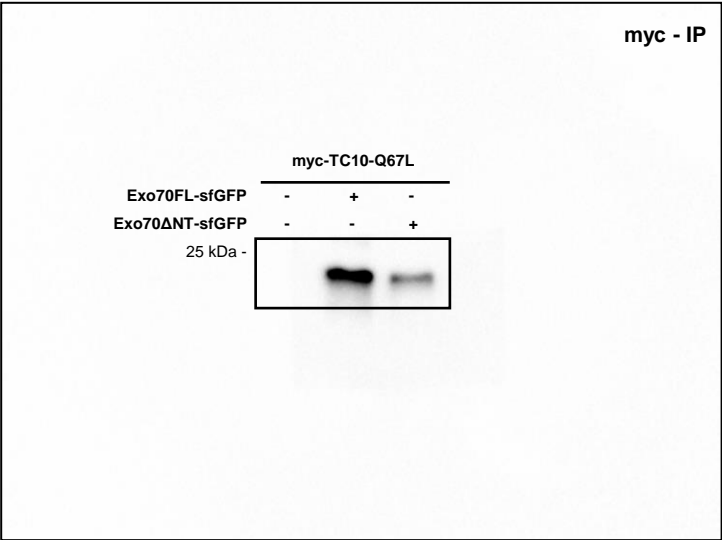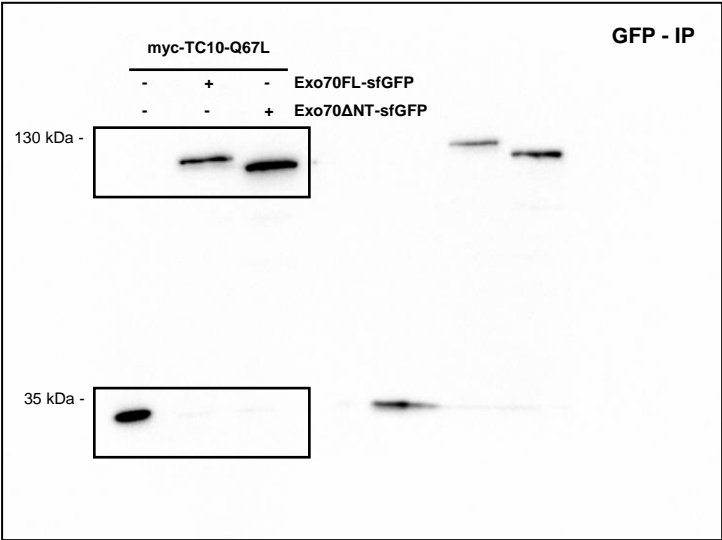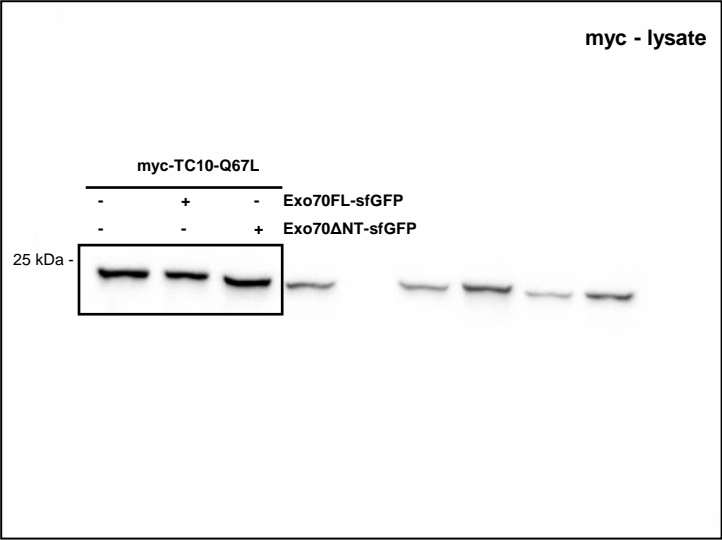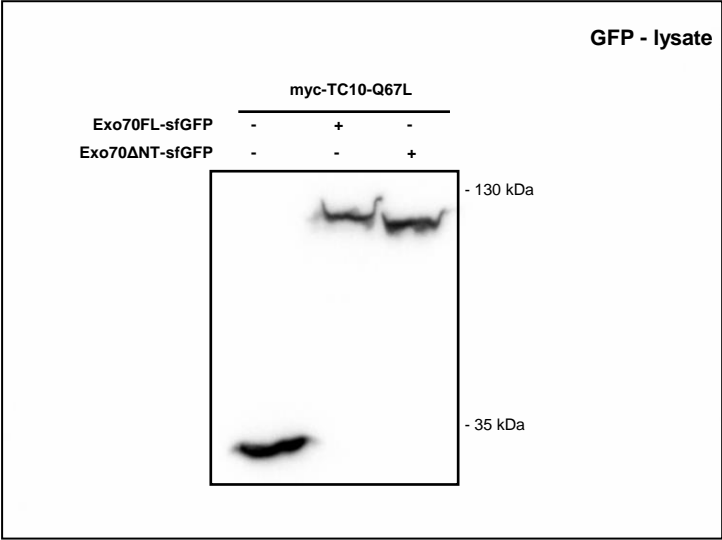

Figure 2F

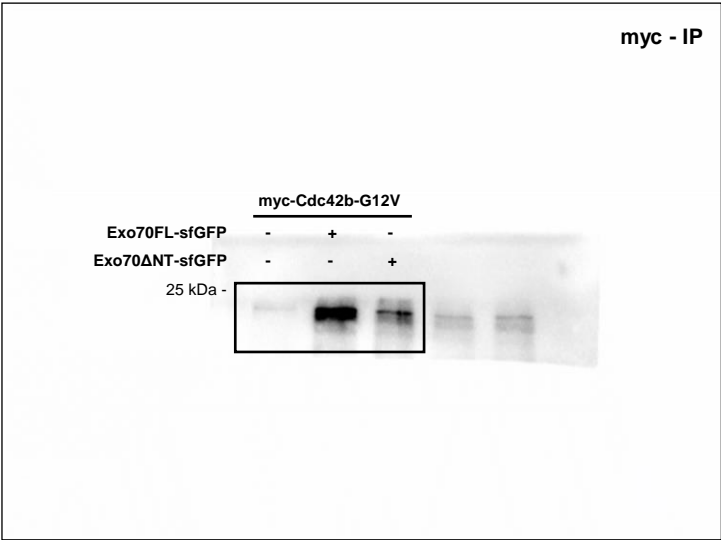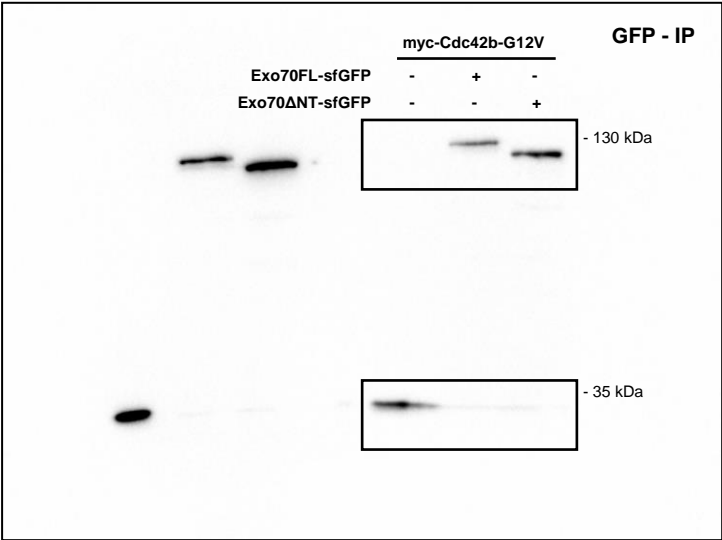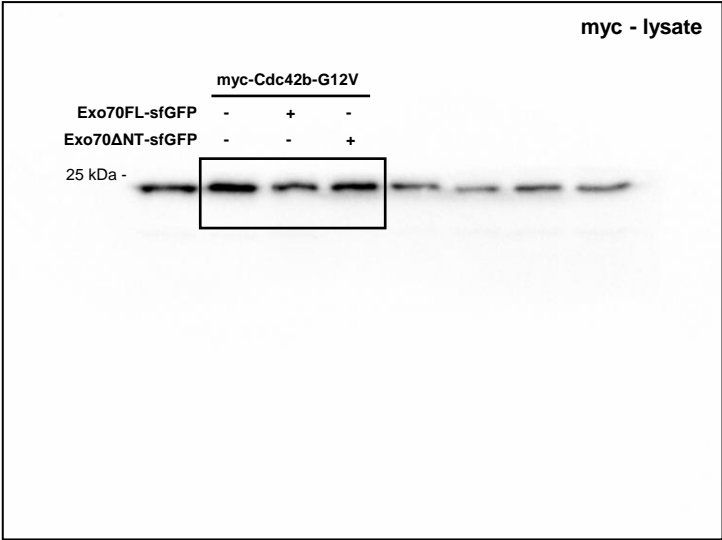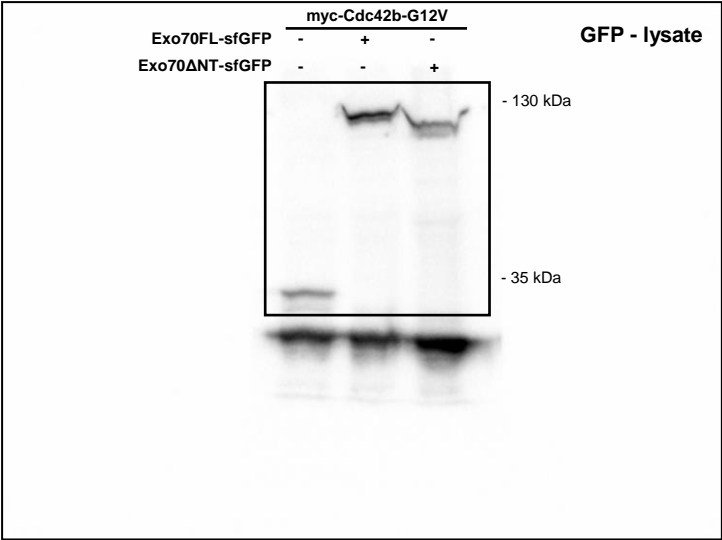

Supplement: Supplementary file 2 [file LSA-2022-01722_SdataF2.1.pdf]

Figure S1B

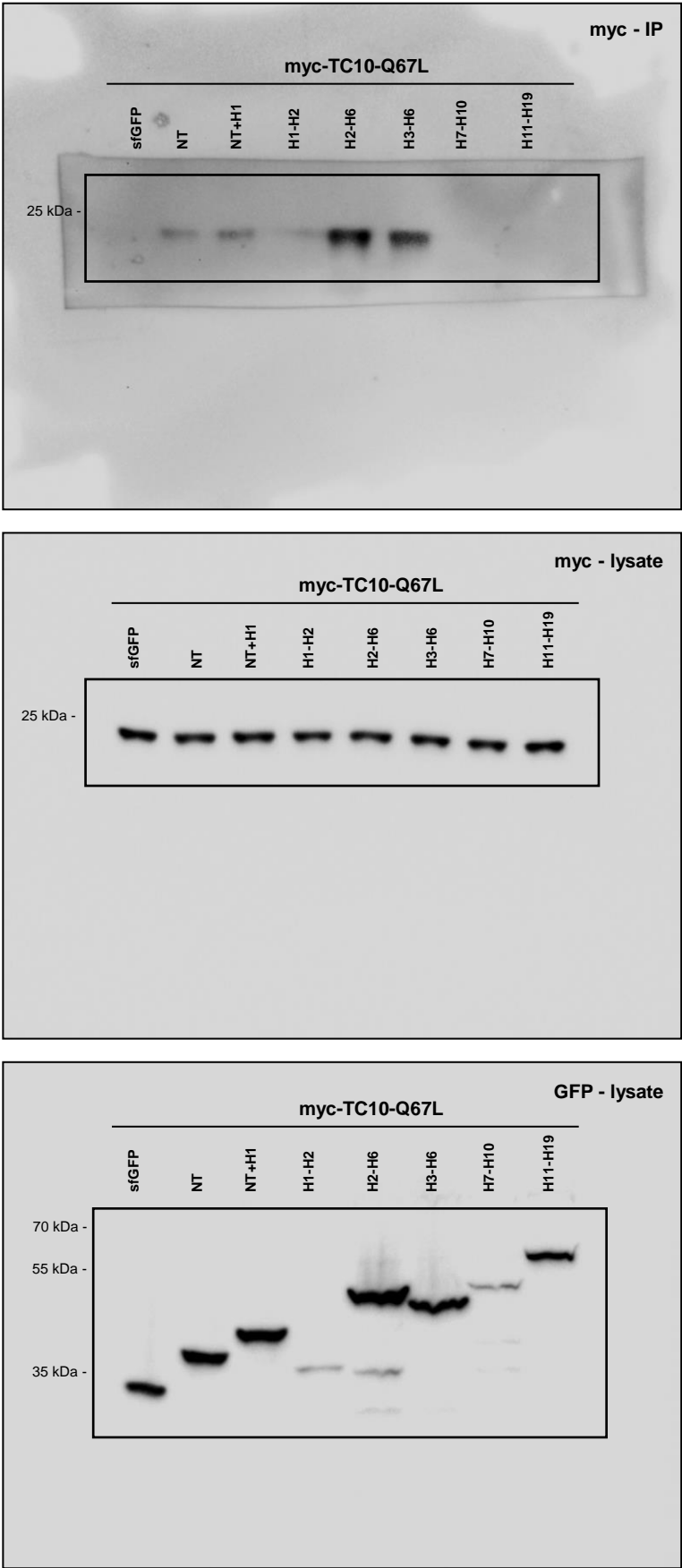

Figure S1C

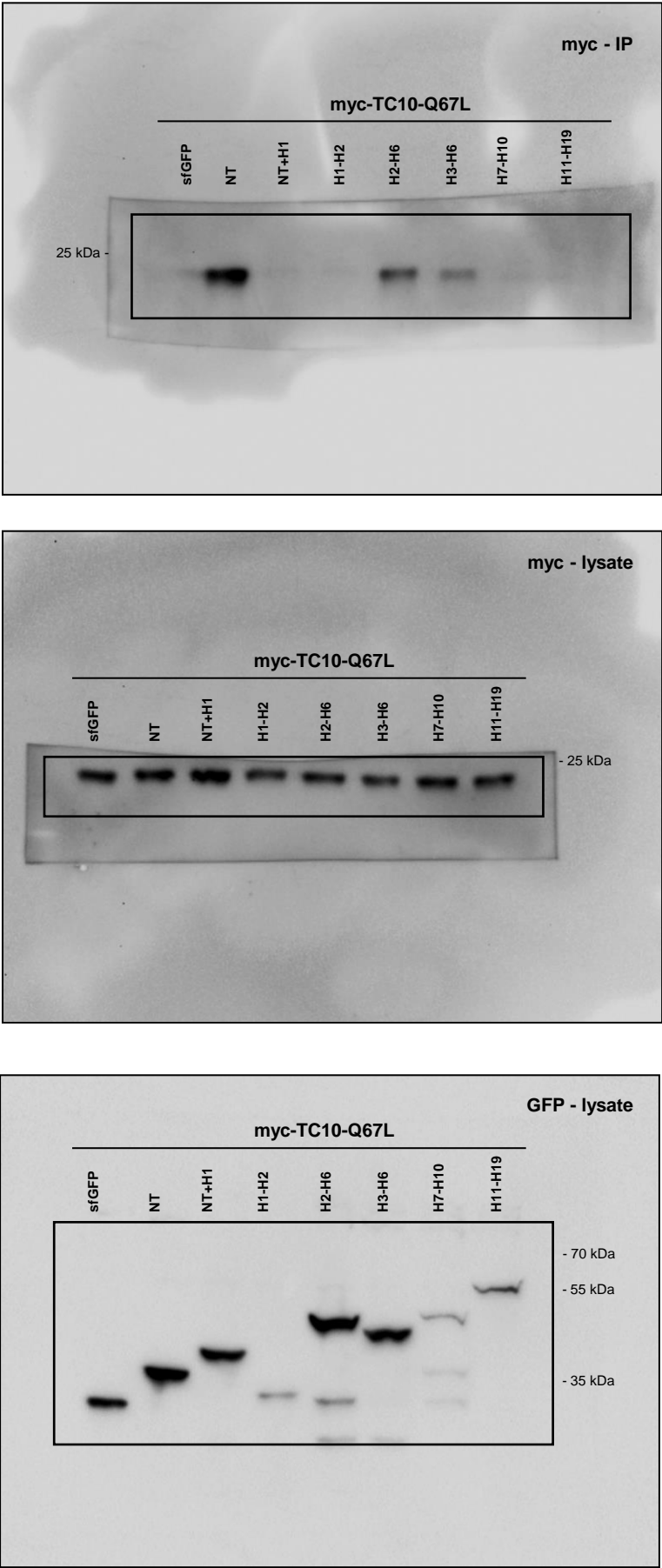

Figure S1D

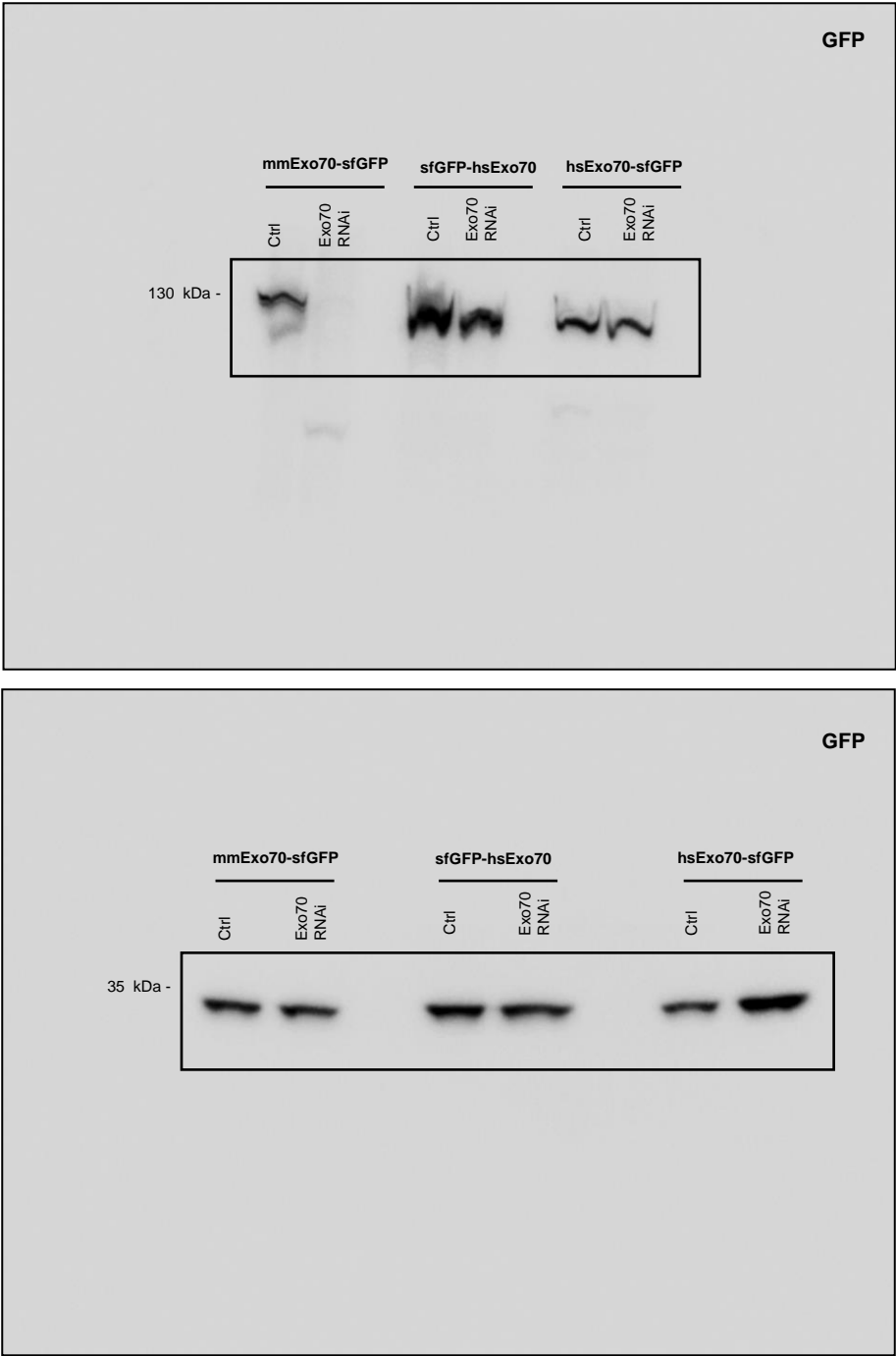

Supplement: Supplementary file 4 [file LSA-2022-01722_SdataFS1.1.pdf]

Figure 3A

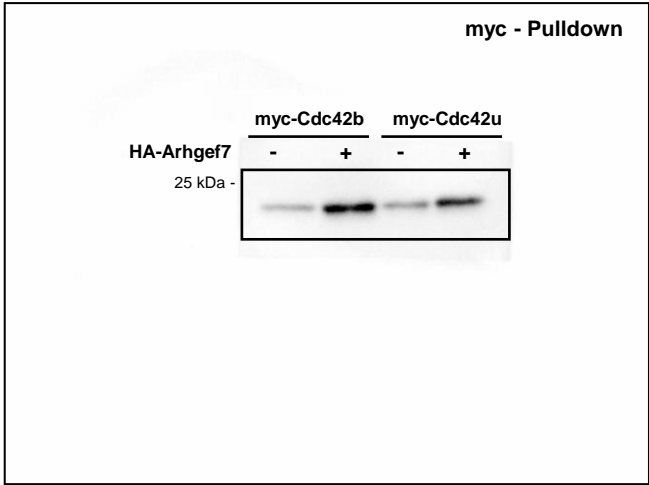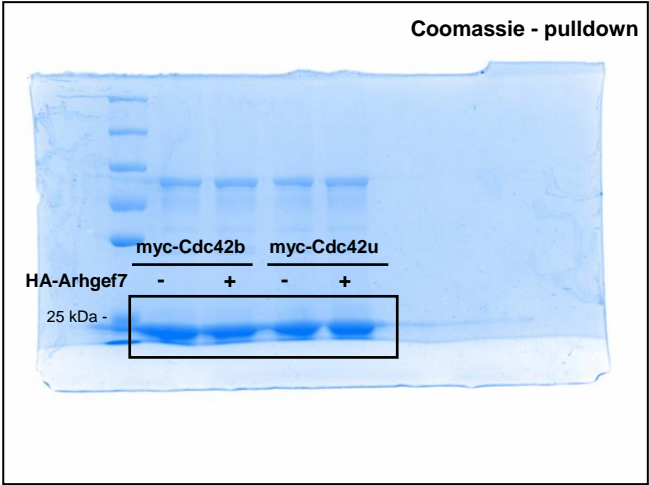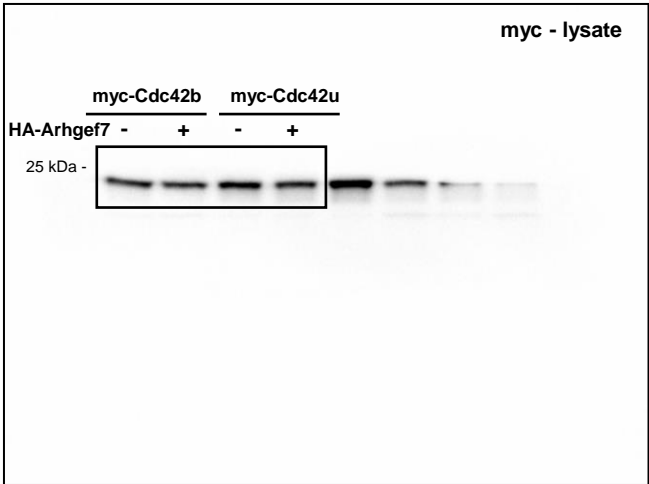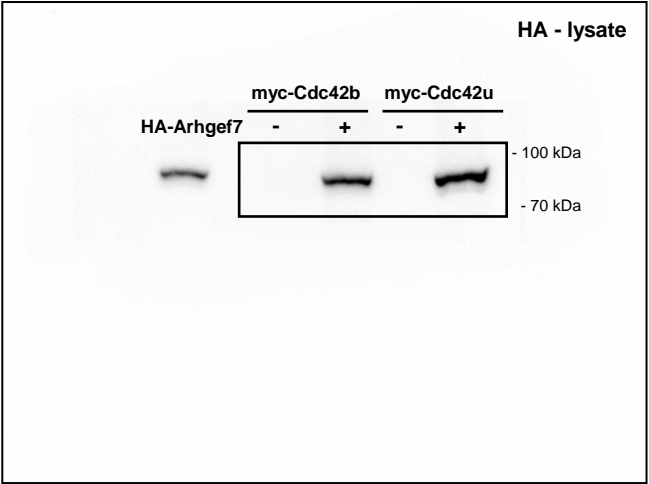

Supplement: Supplementary file 6 [file LSA-2022-01722_SdataF3.1.pdf]

Figure S2A

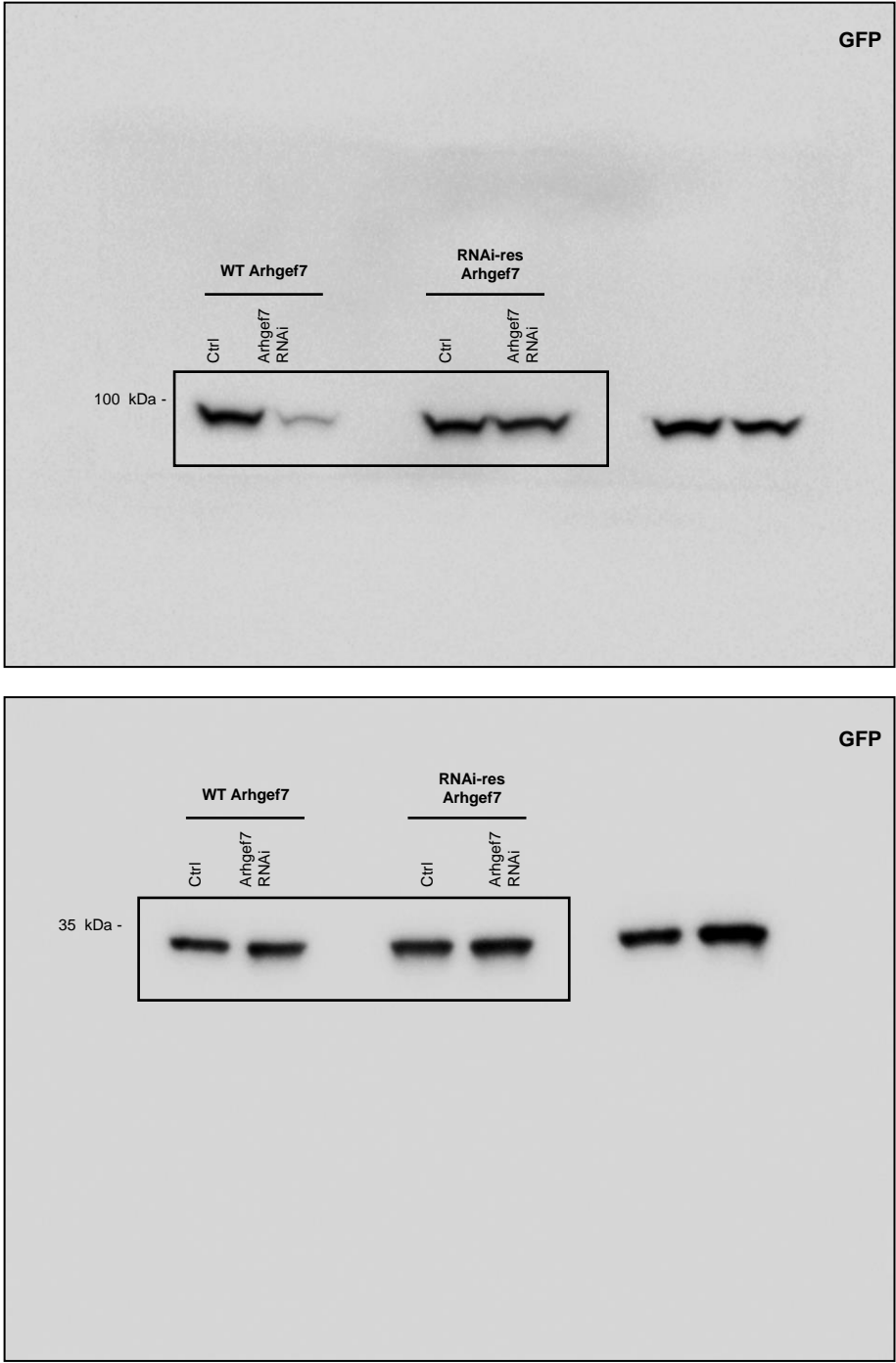

Supplement: Supplementary file 8 [file LSA-2022-01722_SdataFS2.1.pdf]

Figure S3A

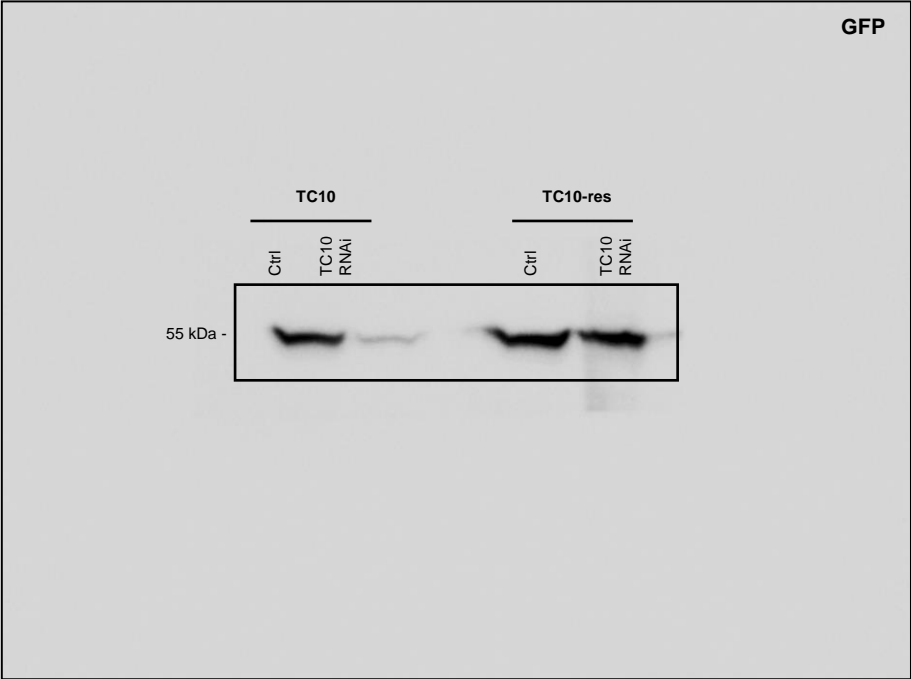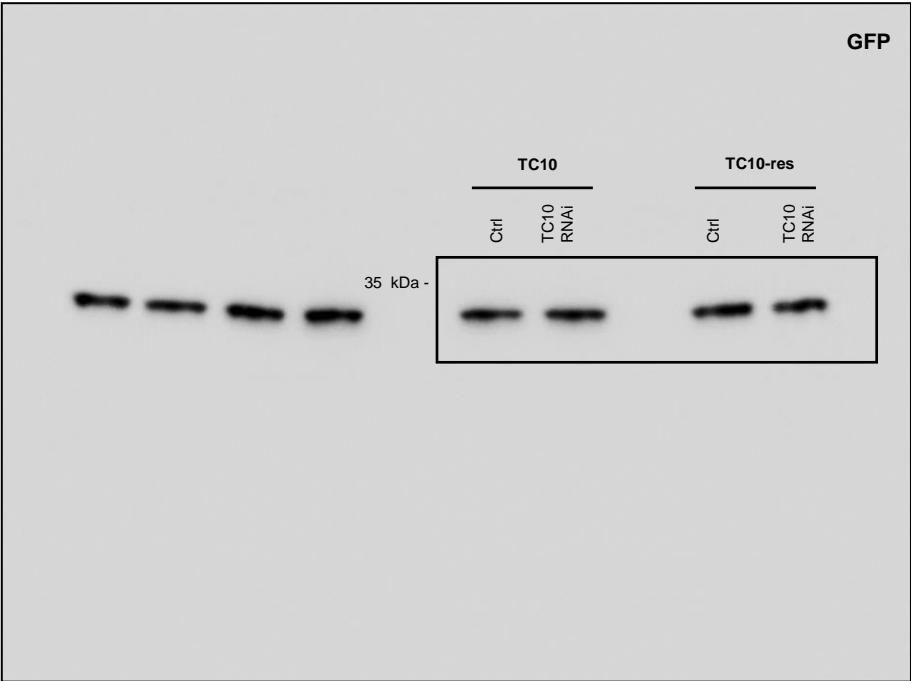

Figure S3E

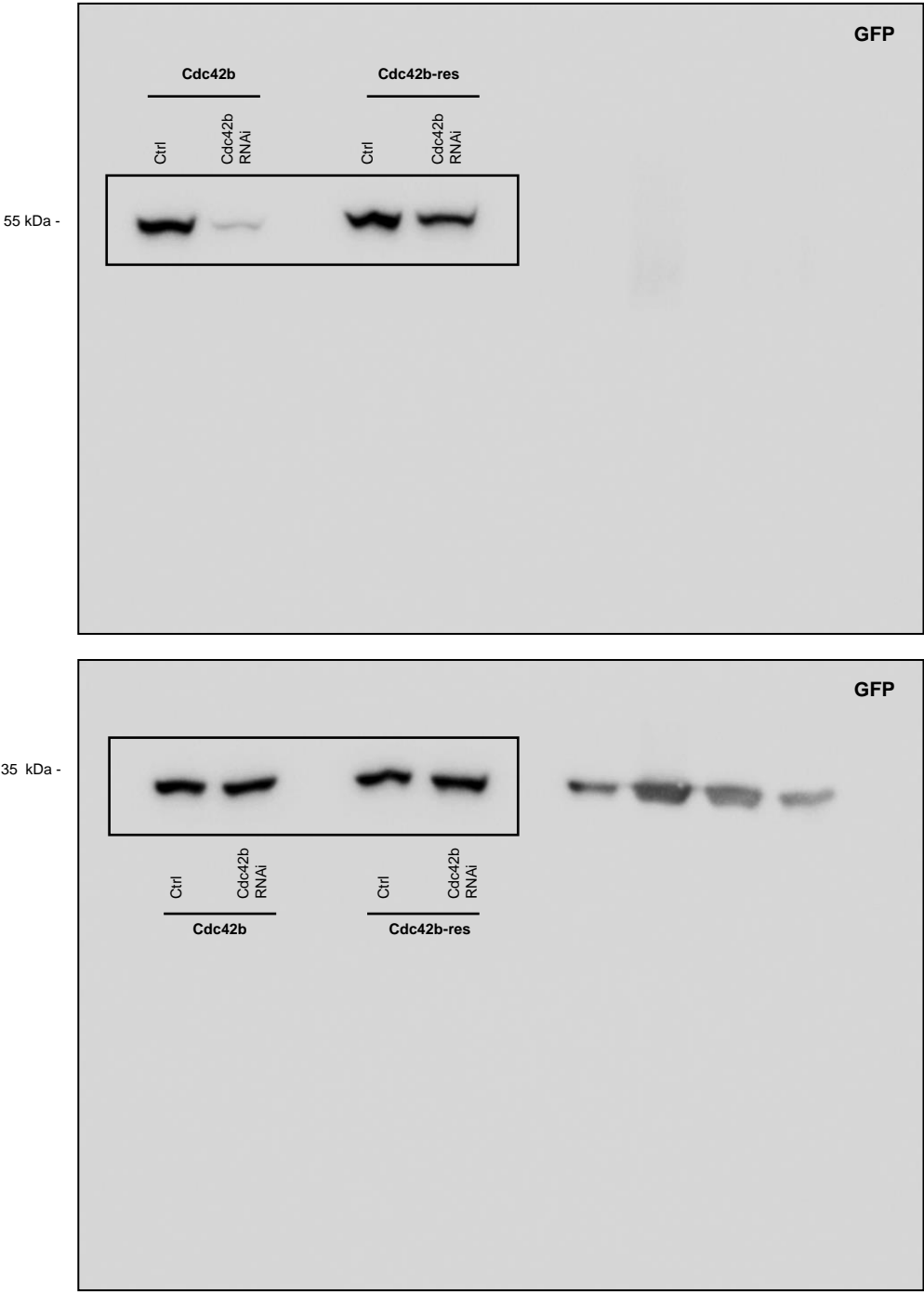

Figure S3G

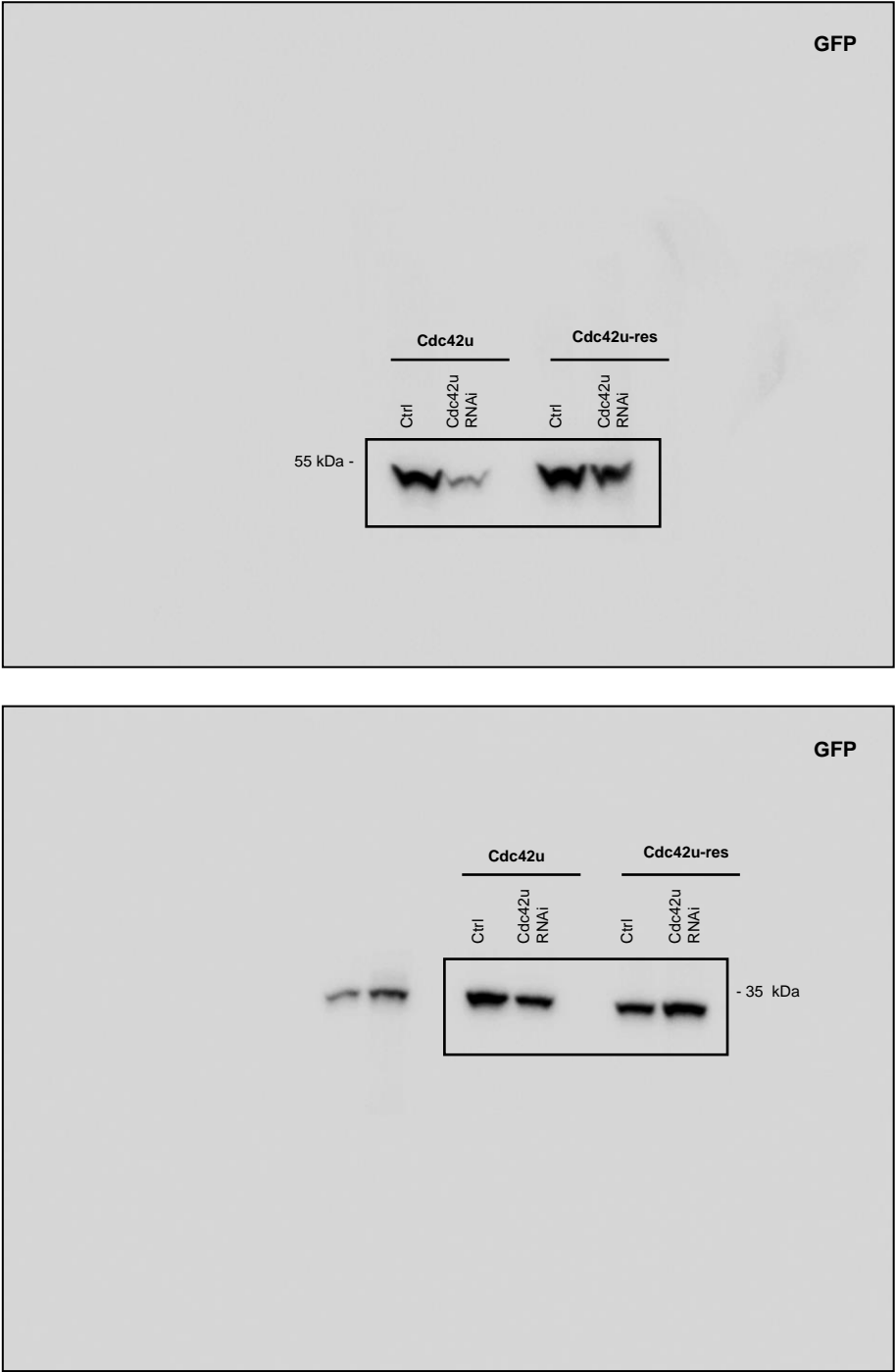

Figure S3I

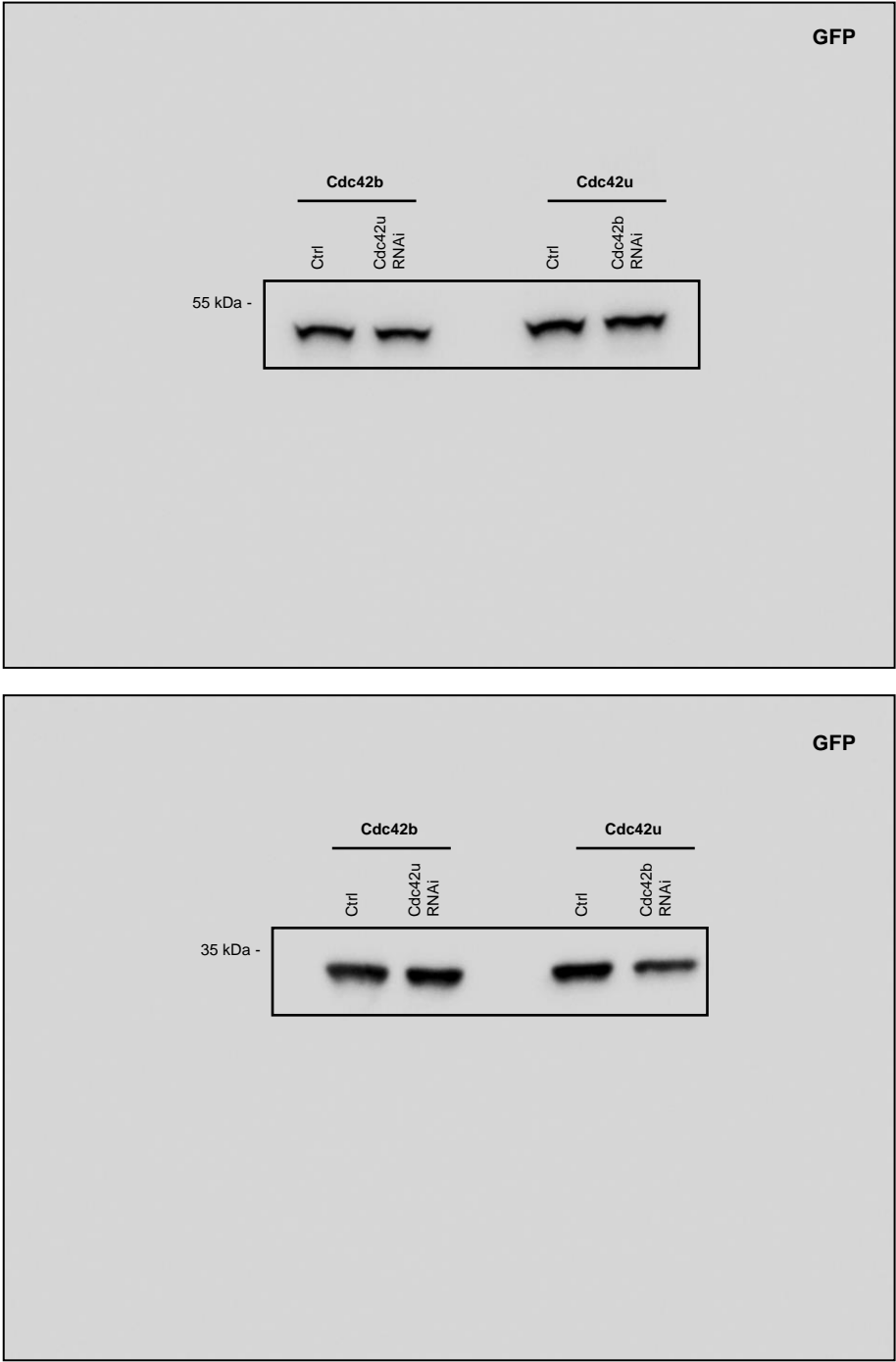

Supplement: Supplementary file 10 [file LSA-2022-01722_SdataFS3.1.pdf]
