# Supplementary material for: An isoform-specific function of Cdc42 in regulating mammalian Exo70 during axon formation
Source: Life Sci Alliance. 2022 Dec 21;6(3):e202201722. doi: 10.26508/lsa.202201722 (PMC9772827; doi:10.26508/lsa.202201722)
Supplement: Supplementary file 13 [file LSA-2022-01722_TableS1.docx]

| **Table S1: Oligonuceotides** | |
| --- | --- |
| **Primer name** | **Sequence 5’-3’** |
| sFGFP-C3 reverse | CAGATCTGAGTACTTGTACAGCTCGTCCATGC |
| mmExo70-sfGFP forward | CGGAATTCTGATGATTCCCCCGCAGGAGG |
| hsExo70-sfGFP forward | CGGAATTCTGATGATTCCCCCACAGGAGG |
| Exo70-sfGFP reverse | GCGTCGACACGGCAGAGGTGTCGAAAAGGCG |
| Exo70 NT reverse | gcGTCGACTTACAGGCAGGACAGCGTCTTCTC |
| Exo70 NT+H1 reverse | gcGTCGACTAATGTGGGGCCCTCTCTGATG |
| Exo70 H1-H2 forward | cgGAATTCTGATGGACCATGTCATCAGCTACTACC |
| Exo70 H1-H2 reverse | gcGTCGACTTAGTCTGGGCTGTTGTCCTGG |
| Exo70 H2-H6 forward | cgGAATTCTGATGGGTAGGCTGGAAGAGTACCTGG |
| Exo70 H3-H6 forward | gcGAATTCtgatgAGCCCGGAACTCAACAAAGTG |
| Exo70 H2-H6 reverse | gcGTCGACTAACTTATGGAAATGCTCCTTC |
| Exo70 H7-H10 forward | cgGAATTCTGATGAGCAGTTCTTCCTCTGGGG |
| Exo70 H7-H10 reverse | gcGTCGACTAAATTCTTGATGTTGTCTGC |
| Exo70 H11-H19 forward | cgGAATTCTGATGGACCCGGACAAGGAGTAC |
| Exo70 miRNA Top strand | TGCTGTCAAACAGCAGCTTCACTTTGGTTTTGGCCACTGACTGACCAAAGTGACTGCTGTTTGA |
| Exo70 miRNA Bottom strand | CCTGTCAAACAGCAGTCACTTTGGTCAGTCAGTGGCCAAAACCAAAGTGAAGCTGCTGTTTGAC |
| Arhgef7 miRNA Top strand | TGCTGATAACCTTCAGGATCTGAGCGGTTTTGGCCACTGACTGACCGCTCAGACTGAAGGTTAT |
| Arhgef7 miRNA Bottom strand | CCTGATAACCTTCAGTCTGAGCGGTCAGTCAGTGGCCAAAACCGCTCAGATCCTGAAGGTTATC |
| TC10 miRNA Top strand | TGCTGAACACAGTCTTCAAACCCTTCGTTTTGGCCACTGACTGACGAAGGGTTAAGACTGTGTT |
| TC10 miRNA Bottom strand | CCTGAACACAGTCTTAACCCTTCGTCAGTCAGTGGCCAAAACGAAGGGTTTGAAGACTGTGTTC |
| Cdc42b miRNA Top strand | TGCTGTTTGGGTTGAGTTTCCGGAGGGTTTTGGCCACTGACTGACCCTCCGGACTCAACCCAAA |
| Cdc42b miRNA Bottom strand | CCTGTTTGGGTTGAGTCCGGAGGGTCAGTCAGTGGCCAAAACCCTCCGGAAACTCAACCCAAAC |
| Cdc42u miRNA Top strand | TGCTGCACACCTGCGGCTCTTCTTCGGTTTTGGCCACTGACTGACCGAAGAAGCCGCAGGTGTG |
| Cdc42u miRNA Bottom strand | CCTGCACACCTGCGGCTTCTTCGGTCAGTCAGTGGCCAAAACCGAAGAAGAGCCGCAGGTGTGC |
| Arhgef7 RNAi rescue forward | GCCCGTGTCACCCAACTCAGGGACATTGAAGAGCCCTC |
| Arhgef7 RNAi rescue reverse | GAGGGCTCTTCAATGTCCCTGAGTTGGGTGACACGGGC |
| TC10 RNAi rescue forward | GCTTTAACCCAGAAGGGCTTGAACACTGTGTTTGATGAGG |
| TC10 RNAi rescue reverse | CCTCATCAAACACAGTGTTCAAGCCCTTCTGGGTTAAAGC |
| Cdc42b/u forward | CGGGATCCATGCAGACAATTAAGTGTG |
| Cdc42b reverse | TTGCGGCCGCTTAGAATATACTGCTCTTCCTTTTGGGTTGAGTTTCCGGAGGCTCGAGGGC |
| Cdc42b C188S reverse | TTGCGGCCGCTTAGAATATACAGCTCTTCCTTTTGGGTTGAGTTTCCGG |
| Cdc42b C189S reverse | TTGCGGCCGCTTAGAATATACTGCACTTCCTTTTGG GTTGAGTTTCCGG |
| Cdc42b C188,189S reverse | TTGCGGCCGCTTAGAATATACTGCTCTTCCTTTTGG GTTGAGTTTCCGGAGGCTCGAGGGC |
| Cdc42b RNAi rescue reverse | CCGCGGCCGCTTAGAATATACAGCACTTCCTTTTGGGCTGGGTCTCCGGAGGCTCGAGGGC |
| Cdc42u RNAi rescue reverse | TTGCGGCCGCTCATAGCAGCACACACCTGCGACTGTTGTTCGGTTCTGG |
